# Alternative mRNA Splicing from the Glial Fibrillary Acidic Protein (*GFAP*) Gene Generates Isoforms with Distinct Subcellular mRNA Localization Patterns in Astrocytes

**DOI:** 10.1371/journal.pone.0072110

**Published:** 2013-08-26

**Authors:** Rune Thomsen, Tina F. Daugaard, Ida E. Holm, Anders Lade Nielsen

**Affiliations:** 1 Department of Biomedicine, Aarhus University, Aarhus, Denmark; 2 Laboratory of Experimental Neuropathology, Department of Pathology, The Regional Hospital of Randers, Randers, Denmark; 3 Center for Integrative Sequencing, *i*SEQ, Aarhus University, Aarhus, Denmark; 4 Lundbeck Foundation Initiative for Integrative Psychiatric Research, *i*PSYCH, Aarhus University, Aarhus, Denmark; Rutgers New Jersey Medical School, United States of America

## Abstract

The intermediate filament network of astrocytes includes Glial fibrillary acidic protein (Gfap) as a major component. *Gfap* mRNA is alternatively spliced resulting in generation of different protein isoforms where Gfapα is the most predominant isoform. The Gfapδ isoform is expressed in proliferating neurogenic astrocytes of the developing human brain and in the adult human and mouse brain. Here we provide a characterization of mouse *Gfapδ* mRNA and Gfapδ protein. RT-qPCR analysis showed that *Gfapδ* mRNA and *Gfapα* mRNA expression is coordinately increased in the post-natal period. Immunohistochemical staining of developing mouse brain samples showed that Gfapδ is expressed in the sub-ventricular zones in accordance with the described localization in the developing and adult human brain. Immunofluorescence analysis verified incorporation of Gfapδ into the Gfap intermediate filament network and overlap in Gfapδ and Gfapα subcellular localization. Subcellular mRNA localization studies identified different localization patterns of *Gfapδ* and *Gfapα* mRNA in mouse primary astrocytes. A larger fraction of *Gfapα* mRNA showed mRNA localization to astrocyte protrusions compared to *Gfapδ* mRNA. The differential mRNA localization patterns were dependent on the different 3′-exon sequences included in *Gfapδ* and *Gfapα* mRNA. The presented results show that alternative *Gfap* mRNA splicing results in isoform-specific mRNA localization patterns with resulting different local mRNA concentration ratios which have potential to participate in subcellular region-specific intermediate filament dynamics during brain development, maintenance and in disease.

## Introduction

Glial fibrillary acidic protein (Gfap) is a component of the intermediate filaments in astrocytes together with Vimentin and Nestin [1]. The intermediate filaments (diameter 8–12 nm) have an important function for signal transduction and structural properties of astrocytes and form the cellular cytoskeleton together with microtubules (diameter 25 nm) and actin microfilaments (diameter 7 nm) [2], [3]. The intermediate filament proteins have a well conserved central helical rod domain involved in filament assembly through dimerization and multimerization and head and tail domains of variable size and amino acid sequence [2]. The head and tail domains can influence assembly of filaments. Gfap and Vimentin are classified as type III intermediate filament proteins due to their capacity to assembly into both homomeric and heteromeric intermediate filaments whereas Nestin belongs to type IV which requires heteromeric intermediate filament proteins for filament assembly [3], [4].

In humans at gestational week 9–12 Gfap expression starts in the radial glial cells [5], [6], [7]. Radial glial cells are bipolar cells in the ventricular zone (VZ) which express Nestin and Vimentin and have neuronal stem cell potential [8], [9]. During the second half of gestation Gfap expression increases and also becomes evident in the arising subventricular zone (SVZ) which persists into adulthood [5], [7], [10], [11], [12], [13]. Gfap expression increases during the maturation and differentiation of the precursor cells whereas Nestin and Vimentin expression decreases. Some astrocytes maintain co-expression of Vimentin and Gfap [14], [15]. Gfap expression is induced by brain damage and CNS degeneration and is also induced during ageing, and altered Gfap expression is associated with a variety of neurological diseases [16], [17]. Increased expression of Gfap, together with Vimentin and Nestin, and enlargement of astrocytes is indicative of reactive gliosis [18]. Gfap missense mutations in the rod and tail domains are involved in Alexander disease where astrocytes accumulate Gfap containing cytoplasmic aggregates [19]. The differences in Gfap expression can alter the morphology of astrocytes with direct consequences for a variety of astrocyte functions during development and ageing, and also have indirect consequences for other CNS cell types [17].

**Figure 1:**
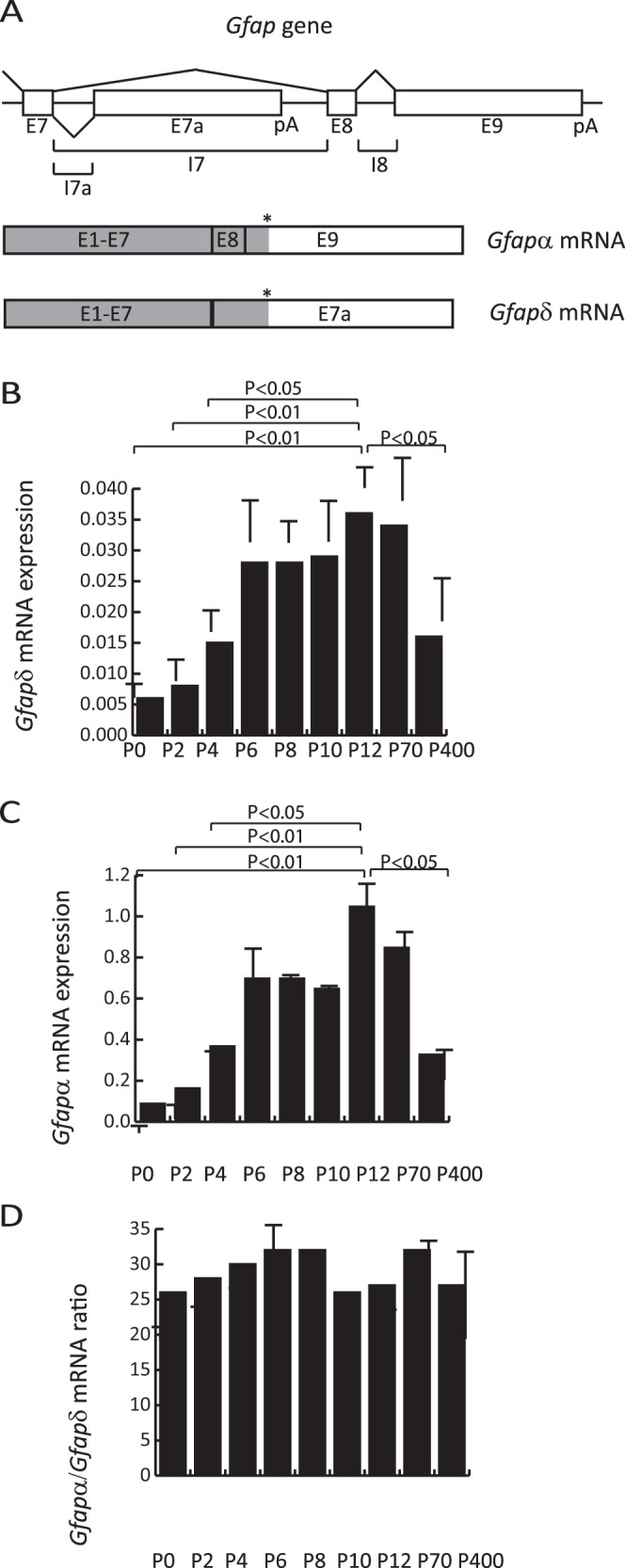
mRNA for the alternative spliced *Gfap* isoform *Gfapδ* is expressed in the postnatal mouse brain. (A) Schematic drawing of the mouse *Gfap* locus. pA indicate positions of poly-adenylation signals in exons E7a and E9. (*) indicate positions of translational stop codons in exons E7a and E9. Coding regions are shown in grey. (B–C) Expression analysis of *Gfapδ* (B) and *Gfapα* (C) mRNA in postnatal mouse brain samples. RNA was isolated from cortices at the indicated time points. cDNA was used in RT-qPCR experiments with primer combinations specific for *Gfapδ* and *Gfapα* cDNA. Results are normalized to the *b2m* mRNA expression level which is determined as reference gene by GeNorm analysis. Results are presented as mean +/− SD. P-values for the expression levels compared to the P12 expression level were calculated by a Student’s unpaired two tailed t-test. The results represent three independent RT-qPCR experiments from one brain sample cohort. Another independent brain sample cohort gave similar results. (D) The post-natal expression ratio between *Gfapα* and *Gfapδ* mRNA is constant. The expression ratio at different time points using the values from panels B and C was calculated and presented as mean +/− SD.

The *Gfap* gene has nine exons and spans 10 kb in the human genome. At least eight different *Gfap* mRNA isoforms exist which are generated as a consequence of alternative mRNA splicing and polyadenylation signal selection [16], [17], [20], [21], [22], [23], [24]. Corresponding Gfap proteins can be expressed in specific astrocyte subtypes and possess the capability to modify the astrocyte intermediate filament network. In humans the most abundant Gfap isoform in the CNS is Gfapα (432 amino acids) [17], [21]. Alternative mRNA splicing combined with alternative polyadenylation of the human *Gfap* gene intron 7 generates a novel exon, E7a, which together with exons 1 to 7 encodes the Gfapδ isoform (431 amino acids) (Fig. 1A) [21], [22], [25]. We previously abbreviated this isoform Gfapε [20], [21], [26] but accept the nomenclature by Middeldorp and Hol and will following use the nomenclature Gfapδ [17], [22], [25]. The mRNA expression level of *Gfapδ* is in the order of 10-fold lower than *Gfapα* [21], [22], [23], [27]. The Gfapδ isoform has a novel tail domain and thereby lacks the capability to assembly into homomeric Gfapδ intermediate filaments but forms heteromeric intermediate filaments with Gfapα and Vimentin [23], [26], [27]. An increased Gfapδ level can result in a Gfap and Vimentin intermediate filament collapse [26], [27]. Moreover, Gfapδ has a specific capacity to form interactions with the Presenilin proteins [21]. Thus, Gfapδ has the potential to function as a modulator of the Gfap filament structure and associations with other proteins, which could influence astrocyte function. In mouse the Gfapδ tail domain is 41 amino acids and 71% homologous to the human Gfapδ tail domain [21], [28]. The rat Gfapδ tail domain is 33 amino acids and 75% homologous to the human Gfapδ tail domain [23], [25], [28]. The degree of homology between mammalian Gfapδ tail domains are lower than for the other Gfap protein sequences [28]. The Gfapδ isoform is only identified in mammals and the alternative exon E7a is proposed to be under a different evolutionary selection pressure than other *Gfap* gene exons [28].

A detailed examination of Gfapδ expression has been performed in the developing human brain showing that at 13–15 weeks of gestation Gfapδ and Gfapα are expressed in parallel in the VZ radial glial cells [11]. From around week 17 of gestation, the neuronal progenitors of the SVZ also express Gfapδ and this expression continues until birth [11]. In human and mouse post-natal Gfapδ expression is observed in a specific subpopulation of astrocytes located along the ventricles in the subventricular zone (SVZ) and in the subpial zone [11], [22], [23], [29]. The SVZ astrocytes express more Gfapδ than astrocytes in adjacent locations and the Gfap expression is highly variable [30]. The Gfapδ positive cells are proposed to constitute neural stem cells of the developing and adult brain [17], [23]. Gfapδ expression is also determined in the rostral migratory stream and the olfactory bulb [23], [30]. In addition, subpopulations of astrocytes express Gfapδ and Vimentin such as fibrillary astrocytes in the molecular layer of the supratentorial cortex and astrocytes of the glia limitans [31]. Together, these studies indicate that in the developing and adult human brain a subgroup of Gfapδ positive cells can represent proliferating neurogenic astrocytes. A recent report showed that all astroglia cells in the developing and adult mouse brain express Gfapδ regardless of the neurogenic potential indicating that in mice Gfapδ is not a neural stem cell marker as in humans [32].

We here extend the characterization of Gfapδ in the mouse brain and describe that the *Gfapδ* and *Gfapα* mRNA isoforms have distinct subcellular localization patterns which indicates a novel mechanism for regulation of Gfap intermediate filament dynamics.

## Materials and Methods

### Ethics Statement

*Gfap(−/−)* and *Gfap(+/+)* mouse tissue slides were a generous gift from Dr. S. Itohara and the animal experiments to prepare these tissue slides [33], [34] have been approved and performed according to the guidelines of the Animal Care, Regulation of Infectious Agents and Experimental Committee, National Institute of Animal Health, Japan.

Killing of experimental animals (for example for collection of organs for further examinations) without prior intervention (surgery, medical treatments, etc.) is not considered an animal experiment and does not require prior approval.

### RNA Purification and RT-qPCR of Brain Samples

Mouse cortices were dissected from P0, P2, P4, P8, P10, P12, P70, and P400 mice. RNA was purified by standard phenol chloroform extraction methods using 1 µg of glycogen for precipitation. cDNA was made using an iScript cDNA synthesis kit (Bio-Rad). 1 µl of total RNA solution was used per reaction. Real time quantitative PCR (RT-qPCR) was performed using SYBR Green 480 master mix (Roche). RT-qPCR analyses were performed using a Roche Lightcycler™ 480 with annealing temperature of 58°C. A common forward primer, *Gfap-all*, F, ACATCGAGATCGCCACCTACA, was used with the following *Gfap* isoform specific reverse primers: *Gfapδ*, R, CCATTTTCAATCTGGTGAGCCTG; *Gfapα*, R, CCTTCACATCACCACGTCCTTG; *Gfapκ*, R, AGATGCATGCCCTAGGATCCT [20], [21], [23]. The primers were designed as intron spanning and PCR primers were determined to have amplification efficiencies close to 100% and specificity and integrity of amplicons were verified by gel electrophorese and melting curve peak analysis. All Ct values (for the presented mouse P0 to P400 brain samples) used for quantitation of transcript levels were within the detection limit. RT-qPCR amplifications were made in triplicates for each gene and the Ct values were converted into linear values using the X_o_ method [35]. Results are presented as mean +/− standard deviation (SD) and represent three independent RT-qPCR experiments. To select for normalization genes in RT-qPCR analysis the expression levels of potential reference genes in the postnatal brain cDNA samples were assessed and used for GeNorm analysis [36]: *Gapdh*, F, GGTGAAGGTCGGTGTGAACG, R, CTCGCTCCTGGAAGATGGTG; *Actb*, F, ACACAGTGCTGTCTGGTGGT, R, CTGGAAGGTGGACAGTGAGG; beta-2-Microglobulin (*b2m*), F, AGACTGATACATACGCCTGCAG, R, GCAGGTTCAAATGAATCTTCAG; *Arpc3*, F, TTTCCTCTCAACGCCATTTA, R, ACCTTCTCACACAGCCTCAG. *b2m* was superior in fulfilling the GeNorm criteria and the presented RT-qPCR data for postnatal brain samples are normalized to the *b2m* mRNA expression level.

### Mouse Primary Astrocyte Cultures, Boyden Chamber Analysis and Direct RNA Sequencing (DRS)

Mouse primary astrocyte cultures were prepared from the cerebral cortex of P0 NMR1 mice (Taconic, Denmark) as previously described [37]. RNA and protein from primary astrocyte protrusions were isolated using a modified Boyden chamber assay as described [37]. The RNA expression levels in the cell protrusion fraction (PF, Boyden chamber membrane underside) and in the cell body fraction (CF, Boyden chamber membrane upper side) of *Actb*, *Gfapκ*, *Gfap*δ and *Gfapα* mRNA were determined by RT-qPCR. The values were used to calculate the relative mRNA localization ratio between PF and CF. *Arpc3* mRNA is not differentially localized in mouse primary astrocytes and the localization ratio was given the value 1 [37]. The calculated mRNA localization ratios were normalized to the *Arpc3* mRNA localization ratio. Results are presented as mean +/− SD. P-values for differences in the localization ratios were calculated by a Student’s unpaired two tailed t-test. All experiments were repeated three times.

RNA isolated from PF and CF pools from three independent Boyden Chamber assays were sequenced by the direct RNA sequencing (DRS) method using the Helicos Biosciences platform (Helicos Biosciences) as previously described [38]. Expression values were processed as RNA transcripts per million reads (tpm). The RNA enrichment in cell protrusions was presented as the relative expression value in PF compared to CF. P-values for DRS mRNA localization data were calculated by Fisher’s exact test.

### FISH

Single RNA FISH was essentially made as described in previous protocols [39]. Briefly, probes consisting of 50-mer single stranded DNA oligonucleotides were synthesized and labeled with Cy3 fluorophore for *Gfapα* FISH analysis or Cy3.5 fluorophore for *Gfap*δ FISH analysis. A total of eight various oligonucleotides were hybridized to each target mRNA. The *Gfap* FISH probes were synthesized by an automated DNA/RNA synthesizer Model 392/394 (Applied Biosystems). Sequences of FISH probes are available in Table S1. Mouse primary astrocytes were seeded onto 18 mm Ø, 0.17 mm thick coverslips (Marienfeld) and cultured as described above. At approximately 60% confluence cells were fixed in 4% paraformaldehyde for 20 min at room temperature, and washed and stored in PBS at 4°C. Before hybridization cells were permeabilized using 0.5% triton X-100 in PBS for 10 min at room temperature, washed in PBS, and then incubated in prehybridization solution, 50% formamide (Sigma; F4761) and 2×SSC (Ambion), for 15 min at room temperature. The probes were hybridized in prehybridization solution supplemented with 2 mg/ml BSA (Roche), 0.2 mg/ml Escherichia coli tRNA (Roche), and 0.2 mg/ml sheared salmon sperm DNA (Sigma; D7656) for 3 h at 37°C. 10 ng DNA probe was used per coverslip. Cells were washed twice with prehybridization solution for 20 min at 37°C, then 10 min in 2 × SSC at room temperature, and in PBS for 10 min at room temperature. Cell nuclei were counterstained with DAPI (0.5 mg/L in PBS). After a final wash in PBS, coverslips were rinsed in double distilled water to remove excess salt, dried and mounted using ProLong gold (Invitrogen).

### Immunohistochemistry

Rabbit monoclonal antibodies were raised against a peptide with a sequence from the mouse Gfapδ tail, GGKSTKEGEGQKVTRPLKRL, on a commercial basis (Thermo Scientific). The antibodies were affinity purified before usage. By western blotting and immunohistochemical the Gfapδ antibody PA2190 was selected for use in the subsequent analysis. We note the presence of a DBA and 129X1 mice SNP which changes the P residue to H in the mouse Gfapδ tail sequence from which the antigen peptide was selected.

Mouse brains from ages P3, P10 and P70 were immersion fixed in formalin, and paraffin-embedded tissue blocks were produced from various brain regions. 10 µm coronal sections were mounted on coated glass slides. The sections were de-paraffinized and boiled in PBS for 25 min to obtain antigen retrieval. The slides were treated with peroxidase block (DAKO) for 5 min, and blocked with bovine serum albumin (BSA) (1 mg/ml) for 10 min. Immunohistochemical analysis were performed using the EnVision+ System-HRP-DAB (DAKO). Pan-Gfap antibody (Polyclonal Rabbit Anti-Gfap, Z0334, Dako) was diluted 1∶3000 incubation 30 min; Nestin antibody (Ms x Nestin, MAB353, Millipore) was diluted 1∶200 incubation 30 min; Vimentin antibody (Mouse monoclonal [VI-10], ab20346, Abcam) was diluted 1∶1500 incubation30 min; Gfapδ antibody (PA2190) was diluted 1∶400 incubation 2 h. The pan-Gfap antibody recognizes both Gfapδ and Gfapα. The brain sections were counterstained with haematoxylen solution. The slides were finally cover slipped with Faramount Aqueous Mounting Medium (DAKO), and analysed by a Leica DM 2500 microscope using Leica IM50 4.0 software. *Gfap(−/−)* and *Gfap(+/+)* mouse tissue slides were a generous gift from Dr. S. Itohara and prepared as described [33], [34]. Briefly, brains from 3 month mice were fixed by intracardiac perfusion with neutral buffered formalin followed by rehydration and embedding in paraffin. Coronal sections (4 µm) including the hippocampal region were used for immunohistochemical staining as described above.

### siRNA and DNA Vector Transfections

For siRNA experiments 1000000 primary mouse astrocytes were immediately before the transfection plated into 10 cm dishes in DMEM with 10% FCS. In 640 µl serum free medium was mixed siRNA to a final concentration of 2 µM and incubated for 5 min. 13 µl Dharmafect was mixed with 1267 µl serum free medium and incubated 5 min. The two solutions were mixed and incubated 20 min, added to the cells, and incubated 24 h. The medium was changed to serum free medium and cells incubated for further 48 h. For Gfapδ depletion we used a mix of two siRNA both targeting the *Gfapδ* 3′-UTR. siRNA sequences (sense): *Gfapδ* (1), GGUUAUACCGAUAGAGCUA(dTdT); *Gfapδ*(2), GAUUCAGCCCAGAGGGUUA(dTdT); non-specific control: AGGUAGUGUAAUCGCCUUG(dTdT) (Eurofins MWG Operon). In the mouse *Gfapδ* NM_001131020 reference sequence *Gfapδ*(1)siRNA target nucleotides 1669–1687 and *Gfapδ*(2) siRNA target nucleotides 2517–2535, both in the 3′-UTR. We note that these siRNA also will target the *Gfapκ* isoform (data not shown) since the entire *Gfapδ* mRNA sequence is included in the *Gfapκ* mRNA [40]. Results are presented as mean +/− SD and represent three independent RT-qPCR experiments. Data were normalized for the *gapdh* expression level or *actb* expression level with similar results (data not shown). The siRNA transfections and examination of mRNA and corresponding protein levels were verified in a biological replicate.

The *Gfap* minigene in the background of pTAG4 was previously described [20]. For each transfection were used 150000 NIH3T3 cells or primary mouse astrocytes cells in 6-well plates using 200 µl serum free medium and 3 µl Xtreme Gene 9 DNA transfection reagent version 03 (Roche). Cells were incubated 24 h before a medium shift to serum free medium and a subsequent incubation for 24 h. The cells were used in a standard Boyden chamber assay with 1.0 µM membranes and RNA was purified from the protrusion fraction (PF) and cell body fraction (CF) from 3 membranes for each transfection and pooled. Primers for RT-qPCR analysis of *Gfapδ* and *Gfapα* chimerical mRNA expressed from pTAG4 were previously described [20]. Results are presented as mean +/− SD and P-values for differences in the localization ratios were calculated by a Student’s unpaired two tailed t-test.

### Western Blotting

A P2 mouse brain extract and protein fractions isolated from the Boyden chamber upper and lower sides were mixed with 5× Loading buffer (Fermentas) and 20× Reducing agent (Fermentas) to a final concentration of 1×. The samples were heated to 95°C for 5 min and centrifuged 1 min at 16000 rpm at room temperature. Samples were loaded onto a Tris-HCl Ready Gel 4–15% (Biorad) and processed at 45 mA until the loading buffer had reached the bottom. Proteins were transferred to a hybond-P membrane (GE Healthcare) at 75V for 30 min at 4°C, and the membrane blocked in 10% skimmed milk powder (Difco) mixed with PBS and 1% Tween 20 (Sigma-Aldrich) for 4 h at room temperature. The membrane was incubated with primary antibodies diluted in PTM buffer (PBS containing 0.5% skimmed milk power and 0.1% Tween 20) ON at 4°C. The membrane was washed in PTM three times and incubated with secondary polyclonal HRP-conjugated antibodies (Dako) diluted 1∶10000 in PTM-buffer 1 h at room temperature, and washed 5× in PTM-buffer. For signal detection was used BM Chemo-luminescence blotting substrate (Roche) and the signal developed (AGFA Curix 60) and monitored with X-ray film (Konica Minolta).

### Immunofluorescence and Double Immunofluorescence

Cells were grown on 18×18×0.17 mm coverslips until 60% confluence, fixed in 4% paraformaldehyde for 20 min at room temperature, washed in PBS and stored in PBS at 4°C. Coverslips were incubated in 0.5% triton X-100 in PBS for 10 min at room temperature followed by several washes in PBS. Blocking was done using 1% BSA in PBS for 30 min at room temperature. Primary antibodies were dissolved in blocking buffer and incubated for 1 h at room temperature. Cells were washed 3 times in PBS and incubating with secondary antibody dissolved in blocking buffer. After 3 washes in PBS double immunofluorescence was performed as described above with a second treatment of primary and secondary antibodies. After final secondary antibody incubation cell were washed 2× and cell nuclei were stained with DAPI, then washed 1× in PBS. Coverslips were rinsed in double distilled water to remove salt, dried, and mounted with ProLong gold. Antibodies used were: Rabbit anti-Gfapδ (PA2190) diluted 1∶100; mouse anti-Vimentin (Abcam; ab20346) diluted 1∶100; and goat anti-Gfapα (GFAP (C-19), sc-6170, Santa Cruz) diluted 1∶100 [11]. Alexa conjugated secondary antibodies (InVitrogen) were diluted 1∶2000.

### Microscopy and Image Processing

All images for FISH and IF analysis were made on a Zeiss axiovert 200 m microscope, with a plan apochromatic 63×1.4 NA objective, a HBO 100 W mercury light source, and a CoolSNAP-HQ cooled CCD camera (Photometrics) operated by MetaMorph®. Filters were from Chroma, Cy3 (41003), FITC (41001), and DAPI (31000), and narrow band pass filters were used for dual labeled RNA FISH Cy3 (SP-102v1) and Cy3.5 (SP-103v1). For RNA FISH we took z-stacks with 20 sections 0.2 µm step size and 500 ms exposure. For detection of RNA on a single molecule resolution, images were processed using the open source software Image J. Background noise was reduced by convolving all frames of a z-stack using a 9×9 gaussian kernel. Z-stacks were collapsed to a 2D maximum intensity projection, and single RNA molecule spots were detected using the “Find Maxima” function in Image J. For the shown immunofluorescence images a single, best in focus, 2D image was selected from a 20 sections z-stack.

## Results

### Characterization of the *Gfapδ* Isoform in Mouse

The mouse *Gfapδ* mRNA isoform from the *Gfap* gene was identified due to the homology to human *Gfapδ* [21] and the mRNA and protein expression was recently described in the mouse brain [23], [32]. A schematic description of the alternative *Gfap* splicing resulting in *Gfapδ* and *Gfapα* is shown in Fig. 1A. To examine *Gfapδ* expression during brain development mouse brain RNA was purified from different developmental times. In brain samples from embryonic mice *Gfapδ* expression was below the cut-off detection limit of the used RT-qPCR quantification method. From P0 to P12 an increased *Gfapδ* mRNA expression was detected (Fig. 1B). Expression was also present at the age of 14 months which was the last time point examined (Fig. 1B). The expression profile of *Gfapδ* was similar to *Gfapα* (Fig. 1C). In accordance, the ratio between the *Gfapα* and *Gfapδ* mRNA expression levels was constant at the examined time points (Fig. 1D). RT-qPCR analysis with different *Gfapδ* and *Gfapα* specific primer combinations systematically indicated an expression level in mouse of *Gfapα* 10-fold to 25-fold higher than *Gfapδ* (data not shown). This is in accordance with previous results [21], [23], [32].

### Gfapδ is Expressed in Mouse Brain Astrocytes with Proximity to Ventricles

**Figure 2:**
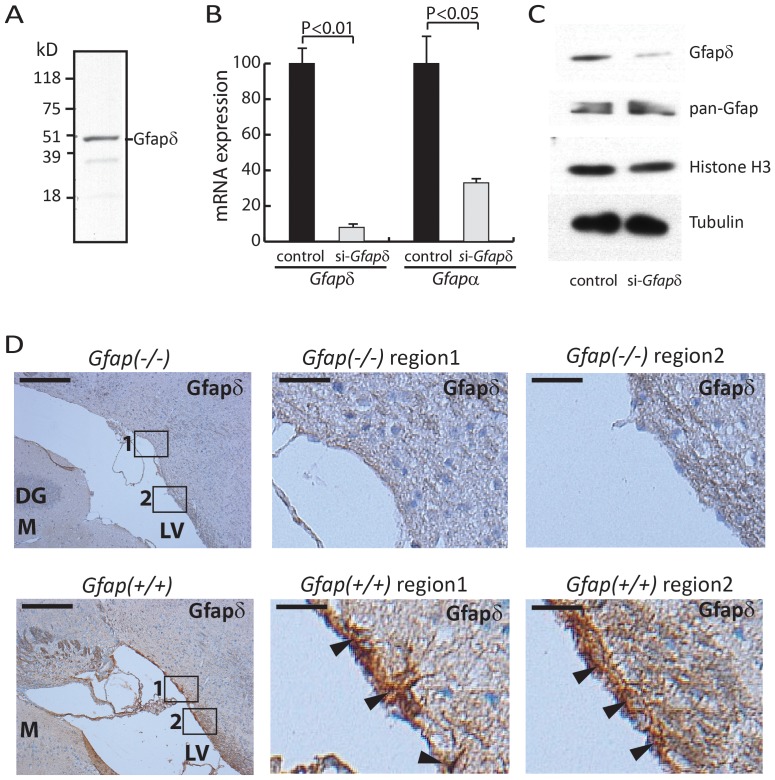
Characterization of mouse Gfapδ. (A) Western blot analysis. A P0 mouse brain extract was analyzed by the Gfapδ antibody. (B) siRNA based characterization of *Gfapδ*. Mouse primary astrocytes were transfected with *Gfapδ* or control siRNA and RNA subsequently purified. Expression of *Gfapα* and *Gfapδ* mRNA was determined by RT-qPCR. Results are presented as mean +/− SD and represent three independent RT-qPCR experiments from material of 3 transfections. P-values were calculated by a Student’s unpaired two tailed t-test. (C) Western-blot analysis of mouse primary astrocytes transfected with *Gfapδ* or control siRNA. The purified protein extract were analyzed with antibodies for Gfapδ and pan-Gfap. For loading controls were used antibodies for histone H3 and Tubulin. (D) Gfapδ antibody specificity confirmation by immunohistochemical staining of *Gfap(+/+)* and *Gfap(−/−)* mouse brain slides. Immunohistochemical staining of formalin fixed *Gfap(−/−)* and corresponding wild type mouse *Gfap(+/+)* brain slides by Gfapδ antibody. The brain slides represent formalin fixed brains from 3 month old mice cut in coronal sections (4 µm) with the hippocampal region shown. The sections were counterstained with haematoxylin solution. DG, Dentate granule cells; M; Dentate molecular layer; LV, lateral ventricle. Right panels represent 10-fold enlargement of the boxed regions 1 and 2 shown in the left panels. Scale bar in left panels 200 µm. Scale bar in right panels 20 µm. Arrowheads in the right panels show Gfapδ staining along the LV in *Gfap(+/+)* mouse brain.

Due to the relatively low conservation of the Gfapδ tail domain between human, rat, and mouse, the cross reactivity of already developed human Gfapδ antibodies has not been satisfactory for immunocytochemically based analysis. To monitor the expression of Gfapδ protein we raised polyclonal antibodies against a peptide sequence corresponding to a mouse Gfapδ tail specific sequence. Western blotting analysis of mouse brain extracts with the antibody PA2190 showed reactivity towards protein with the expected Gfapδ size (Fig. 2A). Other generated antibodies showed similar recognition patterns in mouse brain protein extracts but the PA2190 Gfapδ antibody was used in subsequent analysis due to highest sensitivity. Mouse primary astrocytes were transfected with siRNA targeting *Gfapδ* mRNA, which resulted in a ten-fold reduction of the *Gfapδ* mRNA expression (Fig. 2B). We note a significant down regulation of *Gfapα* mRNA level, which could be explained by the siRNA complementarity to the *Gfapα* intron 7 (Fig. 2B). Notably, we observed reduced Gfapδ protein amounts upon siRNA treatment supporting the specificity of the Gfapδ antibody PA2190 (Fig. 2C). However, pan-Gfap Western blotting showed no significant decrease in Gfap protein despite the observed reduction in also *Gfapα* mRNA (Fig. 2B and 2C). For further control of Gfapδ antibody specificity we performed immunohistochemical staining on brain tissue slides from *Gfap* knock-out mice, *Gfap(−/−),* and corresponding *Gfap(+/+)* control with same genetic background (Figure 2D). Gfapδ staining that was seen in some cells close to ventricles in the wt mouse was absent in the *Gfap(−/−)* mouse (Fig. 2D). The observed staining at the cellular level was in accordance with staining of intermediate filament structures (Fig. 2D). Altogether these results supported that the used immunohistochemical staining protocol was specific for Gfapδ.

**Figure 3:**
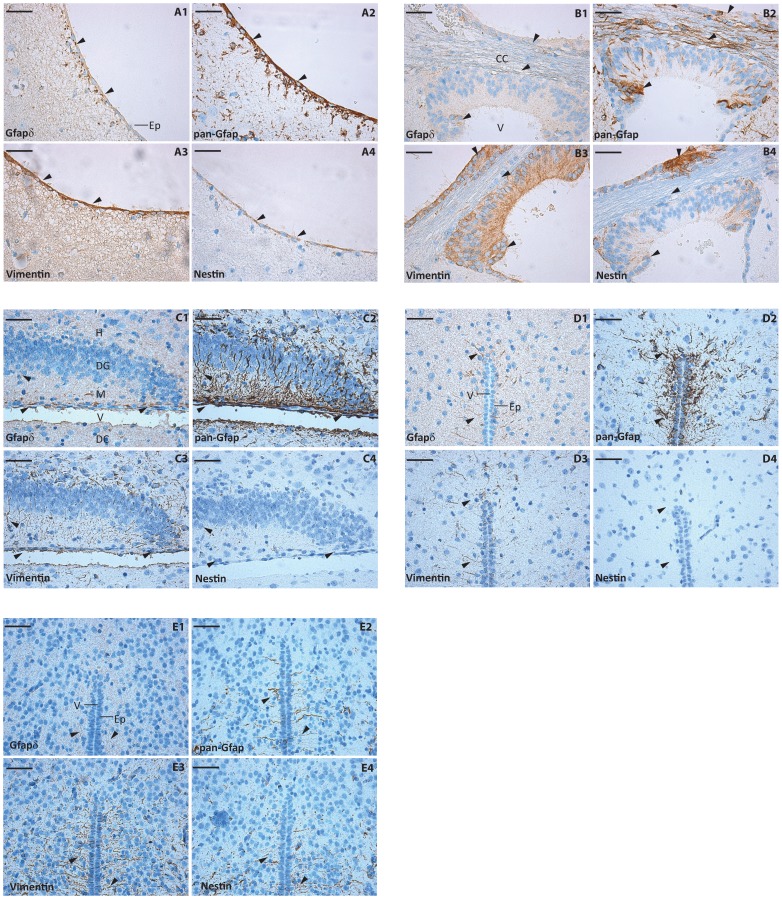
Immunohistochemical staining of Gfapδ in the mouse brain. (A–B) Immunohistochemical staining of mouse P70 brain for Gfapδ (1), pan-Gfap (2), Vimentin (3) and Nestin (4). The sections were counterstained with haematoxylin solution. Upper section panels (A) illustrate lateral ventricle and lower section panels (B) the roof of 3rd ventricle. Ep, Ependymal lining; V, 3rd ventricle; CC, corpus callosum. (C–D) Immunohistochemical staining of mouse P10 brain. Upper section panels illustrate hippocampus (C) and lower section panels brain stem (D). Experimental settings were similar to panels in A–B. Ep, Ependymal lining; V, 4rd ventricle; H, Dentate Hilus; DG, Dentate granule cells; M; Dentate molecular layer; LV, lateral ventricle; DC, Diencephalon. (E) Immunohistochemical staining of mouse P3 brain. Panels illustrate brain stem. Experimental settings were similar to panels in A–B. Ep, Ependymal lining; V, 4rd ventricle. In all Gfapδ panels arrowheads indicate representative Gfapδ antibody stained cells and the corresponding regions also indicated by arrowheads in the pan-Gfap, Vimentin and Nestin stained sections. For all panels scale bar 20 µm.

We performed a more detailed immunohistochemical analysis on mouse brain tissue from P3, P10 and P70. Consecutive brain slides were stained for Gfapδ, pan-Gfap, Vimentin, and Nestin expression (Fig. 3). The overall pattern of Gfapδ staining appeared as few positive cells in the P70 and P10 brains whereas Gfapδ immune reactivity was hardly detected in the P3 brain. In P70 and P10 brains most of the detected Gfapδ positive cells were present in regions with proximity to ventricles or directly along ventricles in a location corresponding to the SVZ (Fig. 3). The distribution of the Gfapδ positive cells along the ventricles was rather non-uniform (Fig. 3). The overall Gfapδ staining pattern was relatively similar at P70 and P10 (Fig. 3). Sections from P70 and P10 were stained with a pan-Gfap antibody which primarily targets Gfapα according to the highest absolute expression of this isoform. The pan-Gfap antibody also recognizes Gfapδ and we selected the use of pan-Gfap to visualize total amount of Gfap due to the very low efficiency of tested Gfapα specific antibodies in our immunohistochemical analysis. pan-Gfap showed a more widespread staining pattern than the Gfapδ antibody. The regions positive for Gfapδ were also positive in the pan-Gfap staining (Fig. 3). At P3 a clear pan-Gfap staining was observed (Fig. 3). Vimentin staining was observed at P70, P10 and P3. In the ventricular areas positive for Gfapδ some Vimentin staining was observed in agreement with co-expression in these cells of the intermediate filament proteins (Fig. 3). Nestin staining in the mouse P70, P10, and P3 brains was more restricted than Vimentin and pan-Gfap staining (Fig. 3). Only in a few groups of ependymal and ventricular cell areas Nestin and Gfap expression were overlapping (Fig. 3). In conclusion, the Gfapδ staining pattern in the post-natal mouse brain resembles findings in the adult human brain and supports that Gfapδ positive mouse brain regions also are Gfapα positive [11], [22], [29], [30], [31].

### Immunofluorescence Analysis of Gfapδ in Mouse Primary Astrocytes

**Figure 4:**
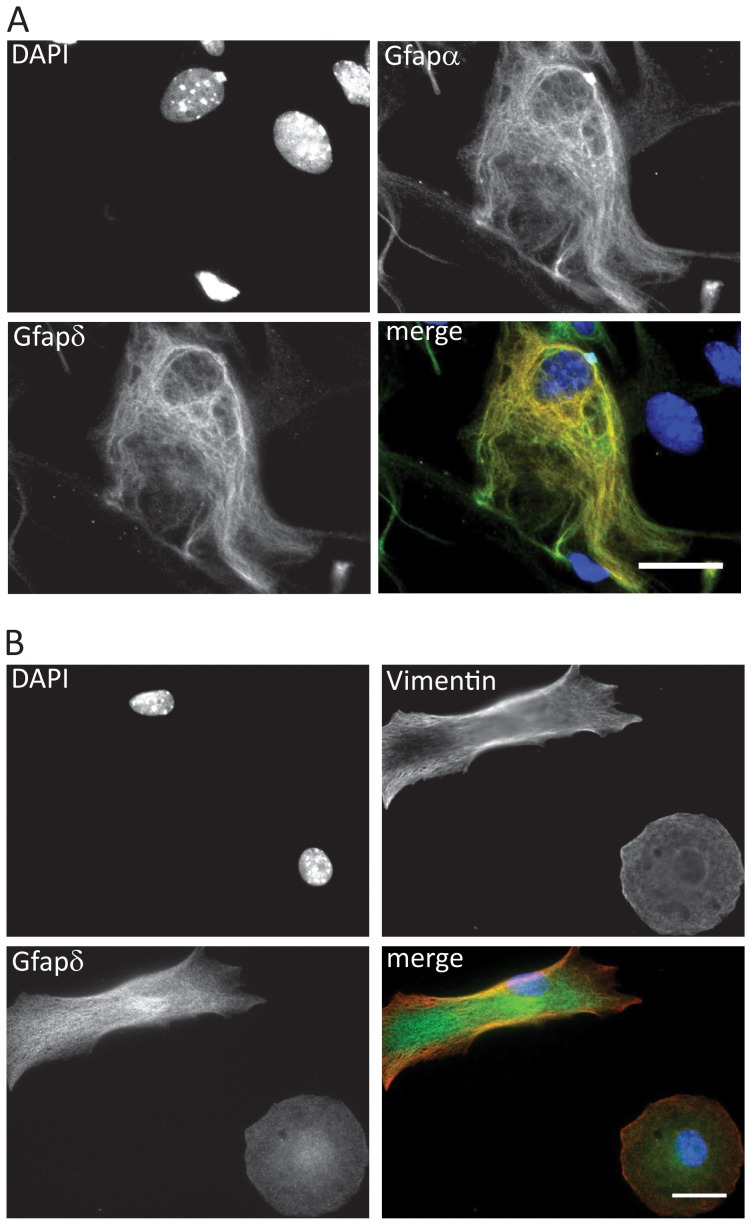
Immunofluorescence analysis of Gfapδ. (A) Co-localization analysis of Gfapα and Gfapδ. Mouse primary astrocytes were stained with primary Gfapδ antibody and Gfapα antibody. The nuclei were counterstained with DAPI. Merged image is included with Gfapα labeled green, Gfapδ labeled red and DAPI labeled blue. (B) Gfapδ and Vimentin have partial co-localization. Mouse primary astrocytes were stained with primary Gfapδ antibody and Vimentin antibody. The nuclei were counterstained with DAPI. Merged image is included with Vimentin labeled red, Gfapδ labeled green and DAPI labeled blue. Scale bar 10 µm.

To examine the subcellular localization of mouse Gfapδ we performed immunofluorescence analysis on cultured primary murine astrocytes. Most of the cells showed a filamentous localization with a Gfapα specific antibody [11]. We used a Gfapα specific antibody instead of pan-GFAP to allow detection of eventual lack in co-localization of Gfapα and Gfapδ. In the fraction of astrocytes with most Gfapα a filamentous Gfapδ localization also was observed (Fig. 4A and data not shown). Co-localization studies showed largely overlapping Gfapδ and Gfapα immunostaining patterns (Fig. 4A). The immunohistochemical analyses (Fig. 3) were pointing towards the existence of some brain regions with co-expression of Vimentin and Gfapδ [11]. Immunofluorescence analysis of the mouse primary astrocytes showed the existence of cells with a filamentous expression of both Gfapδ and Vimentin (Fig. 4B). Co-localization analysis showed only a partial overlap between Vimentin and Gfapδ containing filaments (Fig. 4B). In conclusion, immunofluorescence analysis showed also the presence of Gfapδ in the most Gfapα positive cells supporting that the two *Gfap* mRNA isoforms are transcriptional co-expressed.

### Subcellular mRNA Localization Analyses of *Gfapα* and *Gfapδ*

**Figure 5:**
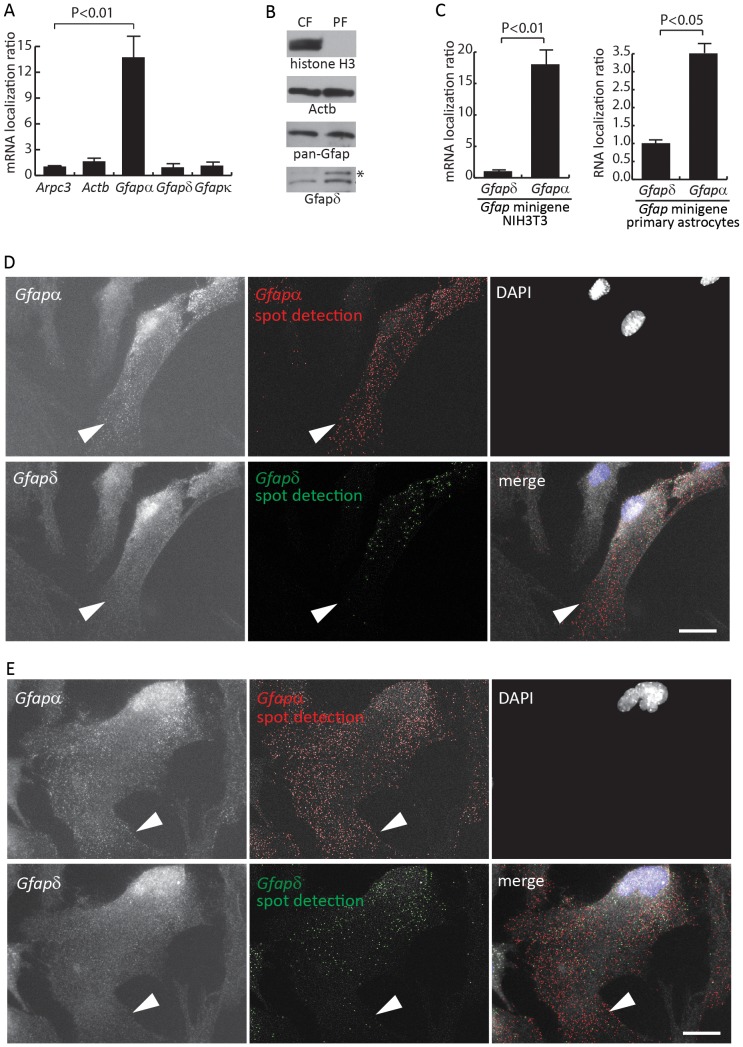
*Gfapα* and *Gfapδ* have isoform specific subcellular mRNA localization. (A) RT-qPCR analysis of the relative localization of *Gfapα, Gfapκ* and *Gfapδ* mRNA from the astrocyte protrusion fraction (PF) and the cell body fraction (CF). The localization ratio is visualized relative to the localization ratio for *Arpc3* mRNA given the value 1. *actb* mRNA localization was also examined. Results are presented as mean +/− SD and represent three independent RT-qPCR experiments from material representing 2 independent Boyden chamber inserts. P-values were calculated by a Student’s unpaired two tailed t-test. (B) Western blotting analysis of Gfapδ, pan-Gfap, Actb and histone H3 protein from astrocyte protrusion fraction (PF) and cell body fraction (CF). (*) indicates an uncharacterized band of approximately 60 kD enriched in protrusions. (C) *Gfap* minigene based mRNA localization analyses. Mouse primary astrocytes and NIH3T3 cells were transfected with a *Gfap* minigene inserted in the pTAG4 vector. Following a Boyden chamber analysis purified RNA samples from protrusions and cell bodies were analyzed by RT-qPCR with primer combinations specific for *Gfapα* and *Gfapδ* mRNA expressed from the pTAG4 minigene and mRNA localization ratios determined by division. Results are presented as mean +/− SD and represent three independent RT-qPCR experiments from 2 independent Boyden chamber inserts. P-values were calculated by a Student’s unpaired two tailed t-test. (D–E) FISH analyses showing representative examples of *Gfapα* and *Gfapδ* mRNA localization in mouse primary astrocytes. The cells were probed with a mixture of 8 *Gfapδ* mRNA Cy3.5 labeled probes and 8 Gfap*α* mRNA Cy3 labeled probes (left panels). FISH images were analyzed for spot detection (central panels) as described in the materials and methods section. The nuclei were counterstained with DAPI. A merged image is shown with *Gfapα* labeled red, *Gfapδ* labeled green and DAPI labeled blue. Arrowheads show astrocyte protrusions and for all panels scale bars represent 20 µm.

Subcellular mRNA localization is mediated by a combination of mRNA *cis*-elements, as for example the Zip-code and G-rich sequences, and associated transport involved protein factors [41], [42]. *Cis*-regulatory elements are often residing in the 3′-UTR, and alternative splicing and polyadenylation consequently generate different coding regions and 3′-UTRs of the *Gfapα* and *Gfapδ* isoforms [21]. Previous studies indicated that a fraction of the *Gfap* mRNA is actively localized to cell protrusions [43]. Therefore, we examined if mRNA localization could be different between *Gfapα* and *Gfapδ*. We have recently described a modified Boyden chamber based method to isolate mRNA present in astrocyte protrusions [37]. The mRNA present in protrusions from mouse primary astrocytes was isolated from the underside of the Boyden chamber membrane, whereas total cellular mRNA was represented by cell material from the membrane upper side. By RT-qPCR we examined for *Gfapδ* and *Gfapα* mRNA accumulation in the protrusion fraction (PF) and compared it to the entire cell represented by the Boyden chamber upper side cell body fraction (CF). The primer combinations described in Fig. 1 were used for the RT-qPCR analysis. In addition, we analyzed the expression of *Actb* mRNA and *Arpc3* mRNA where the latter have uniform distribution in the mouse primary astrocyte cytoplasm [37]. Relative to *arpc3* mRNA, a 13-times increase in *Gfapα* mRNA accumulation was observed in primary astrocyte protrusions (Fig. 5A). To this end, it is very important to note that only a small fraction of the total amount of *Gfapα* mRNA localizes to the protrusions and the localization ratio indicates the grade of *Gfapα* mRNA enrichment relatively to *Arpc3* mRNA. The localization ratio for *Gfapδ* mRNA was similar to *Actb* and *Arpc3* mRNA (Fig. 5A). This supports that the exon E7a present in *Gfapδ* lacks mRNA localization signals present in the 3′-sequence of *Gfapα* mRNA. The *Gfapκ* mRNA isoform includes the intron 7a sequence compared to the *Gfapδ* mRNA [40] (Fig. 1A) and accordingly *Gfapκ* mRNA also lacks *Gfapα* exon 8 and exon 9 which could contribute to mRNA localization. By RT-qPCR we determined that *Gfapκ* mRNA, similar to *Gfapδ* mRNA, lacked mRNA localization in astrocyte protrusions (Fig. 5A). Thus, the different *Gfap* mRNA isoforms display different mRNA localization patterns in astrocyte protrusions.

We performed a direct RNA sequencing (DRS) of polyadenylated RNA isolated from primary mouse astrocyte protrusions and cell bodies. In this experiment no amplifications of material is introduced and RNA sequences are obtained corresponding to the polyadenylated region. Extracting *Gfap* mRNA data from the DRS experiment again showed a relative increased *Gfapα* mRNA localization to cell protrusions compared to *Gfapδ* (Table 1). It should be noted that the *Gfapκ* isoform share a common mRNA 3′-terminal exon sequence with *Gfapδ*, and therefore sequence reads from both isoforms will be uniformly aligned and counted in the DRS experiment. Notably, *Gfapδ* is expressed to a higher level than *Gfapκ* pointing that *Gfapδ* will be the primary detected isoform in DRS [20], [23], [40].

**Table 1:** Direct RNA sequence analyses of *Gfap* mRNA isoform localization in mouse primary astrocyte protrusions.

Transcript	Accession number	TPm Cell bodies	TPM protrusions	Ratio	P-value
*Gfapα*	NM_010277	6003	30759	5.12	9.4E-31
*Gfapδ*	NM_001131020	581	269	0.46	0.54
*actb*	NM_007393	6921	3849	0.56	1
*arpc3*	NM_019824	600	331	0.55	1
*tuba1a*	NM_011653	5301	6154	1.16	7.0E-4
*rpl13*	NM_016738	2498	3699	1.48	2.4E-6

TPM, transcript number per million sequence reads. The total number of sequence reads was 5444770 for the cell body fraction and 2147050 for the protrusion fraction. Two-tailed p-values were calculated by Fisher’s exact test.

Protein expression analyses of cell fractions from protrusions (PF) and cell bodies (CF) from Boyden chamber experiments showed the same relative distribution of Actb, Gfapδ and Gfapα (Fig. 5B). Thus, the differences in *Gfap* mRNA localization were not equivalently reflected in major differences in protein localization patterns. This was in accordance with only a minor amount of the total *Gfapα* mRNA population specifically localizing to protrusions.

To determine if the different compositions of 3′-exons in *Gfapδ* and *Gfapα* were involved in mediating the localization of *Gfapα* mRNA to cell protrusions we performed cell transfection analyses with a *Gfap* minigene including the *Gfap* gene sequences from exon 6 to 1000 bp downstream the exon 9 located polyadenylation signal used for generation of *Gfapα* mRNA [20]. Mouse primary astrocytes were transfected with the minigene. Following a Boyden assay RNA was purified from protrusions and cell bodies and synthesized cDNA examined by RT-qPCR. The ectopic mRNA was measured by a common forward primer recognizing chimeric exon sequences from the minigene vector pTAG4 and reverse primers specifically for *Gfapδ* or *Gfapα*. Calculation of mRNA localization ratios showed 3.5-fold increased *Gfapα* mRNA localization relatively to *Gfapδ* (Fig. 5C). We observed a systematically inefficient transfection efficiency of mouse primary astrocytes with the *Gfap* minigene and accordingly also performed the localization experiment in a readably transfectable mouse cell line. For this we transfected the *Gfap* minigene in mouse NIH3T3 cells which previously were used as model in mRNA localization studies [41]. In this cellular background we observed 20-fold increase in *Gfapα* mRNA localization relatively to *Gfapδ* (Fig. 5C). From the minigene transfection experiments we conclude that the *Gfapα* specific 3′-exons include sequence determinants for *Gfapα* mRNA localization.

The RNA localization studies were extended by single molecule RNA fluorescence *in situ* hybridization (FISH) analysis. Primary astrocytes were analyzed simultaneous with 8 *Gfapα* and 8 *Gfapδ* mRNA FISH probes labeled with Cy3 and Cy3.5, respectively. The FISH probe sets were designed to have specificity towards each *Gfap* isoform (Table S1). *Gfapδ* mRNA FISH analysis resulted in a relative faint number of total signals (148 and 303 single RNA foci in the representative pictures shown in Fig. 5D and 5E, respectively) and with mRNA signals mostly restricted to the cell soma (Fig. 5D–E). *Gfapα* mRNA FISH analysis resulted in a larger number of signals (808 and 1469 single RNA foci in the representative pictures shown in Fig. 5D and 5E, respectively) dispersed through the cell including the cytoplasm, rim and protrusions (Fig. 5D–E). The Increased appearance of *Gfapα* FISH signals compared to *Gfapδ* supports the presence of a lower amount of *Gfapδ* mRNA compared to *Gfapα* mRNA. Incubating the cells with either *Gfapα* or *Gfapδ* mRNA probes individually gave similar results (data not shown). The FISH analysis further supported the localization of a fraction of *Gfapα* mRNA in astrocyte protrusions in alignment with the Boyden chamber analysis. The low level of *Gfapδ* mRNA hampered significant conclusions from the FISH analyses concerning a relative lower localization ratio of this isoform compared to *Gfapα* mRNA in astrocyte protrusions but were indicative of relative more *Gfapδ* mRNA in the central part of the cells.

## Discussion

We here describe the characterization of the mouse *Gfapδ* isoform at the levels of mRNA and protein. The alternative processing of *Gfap* mRNA which results in generation of the Gfapδ protein isoform is conserved between human and mouse [20], [21], [23], [28]. In the *Gfapδ* transcript the exon 8 and 9 sequences used for generating the *Gfapα* mRNA isoform are skipped whereas a novel exon E7a generated from intron 7 is included [17], [21]. Exon E7a includes a novel polyadenylation signal whereby eliminating the use of the exon 9 located polyadenylation signal used for generation of *Gfapα* [20], [21]. Of notable difference in the *Gfap* gene structure between human and mouse is the presence of repetitive elements immediately after the *Gfapδ* exon E7a stop codon in mouse. The relative mRNA expression level of *Gfapδ* compared to *Gfapα* was previously estimated to be in the order of 10% [21], [23], [27], [32]. Our RT-qPCR analysis and direct sequence analysis are also in support of an mRNA expression ratio of this magnitude for these two *Gfap* isoforms. By screening postnatal mouse brain tissue for *Gfapα* and *Gfapδ* mRNA expression we found a coordinated expression. In embryonic tissue, the mRNA expression level of *Gfapδ* was undetectable with the sensitivity by our used RT-qPCR approach, whereas *Gfapα* expression was significantly detected, but to a relative low level (Data not shown). *Vimentin* and *Nestin* mRNA have expression levels in the mouse brain which gradually decreases post-natal. The *Gfap* expression results are concordant with previous analysis from the human, pig and mouse brains in where *Gfapδ* and *Gfapα* expression emerges from mid-gestation [11], [17], [22], [23], [32], [40].

The low degree of homology between the Gfapδ protein tail domains in mouse and human has hampered the use of antibodies developed to detect the human Gfapδ isoform in mouse tissue. Recently, a mouse Gfapδ antibody was described and Gfapδ expression in the adult and developing mouse brain carefully characterized [23], [32]. We here describe the generation of another antibody detecting mouse Gfapδ. In the characterization of the antibody specificity in *Gfapδ* siRNA transfected mouse primary astrocytes we observed that *Gfapδ* siRNA also was targeting the *Gfapα* mRNA expression level despite only recognizing intron 7 in the *Gfapα* primary RNA. *Gfapδ* and *Gfapα* expression levels were 10-fold and 3-fold down regulated, respectively. A coordinated Gfapδ protein down regulation was observed, but we did not observe such an effect for the Gfapα protein level. An effect of the siRNA for *Gfapα* mRNA expression could be dependent on nuclear events at the level of transcription and RNA processing and thereby affecting the *de novo* generation of *Gfapα* mRNA. This scenario is similar to the described transcriptional gene silencing by intron targeting exogenous siRNA [44]. In addition, the siRNA effect on the *Gfapδ* mRNA level could be on stability and translation of already present *Gfapδ* mRNA at the time of siRNA transfection and thereby have stronger effect on the Gfapδ protein level in the time frame of the siRNA experiment. Possible different protein stabilities of Gfapα and Gfapδ could also participate to the observation. We performed a characterization of Gfapδ expression at the protein level in the developing post-natal mouse brain and at the subcellular level in mouse primary astrocytes. In line with a coordinated expression of Gfapδ and Gfapα the immunofluorescence studies in mouse primary astrocytes showed that all filamentous Gfapδ positive cells also were filamentous Gfapα positive, and that a high Gfapα expression was prognostic for Gfapδ expression. Cells with an intermediate or low Gfapα expression had only faint or no filamentous Gfapδ staining. Gfapδ and Vimentin immunofluorescence studies showed that a subset of the primary astrocytes expresses both proteins, but that only partial co-localization was evident.

Immunohistochemistry studies showed rather identical Gfapδ staining patterns in P10 and P70 mouse brains. Immunohistochemistry analysis showed that Gfapδ expression in our assays was only at the limit of detection in the P3 mouse brain whereas at P3 we observed Gfapα, Vimentin and Nestin expression. We note that Gfapδ expression recently was described to increase from E18 to P5 and then decrease until plateauing at P25 [32]. One explanation of the faint Gfapδ staining at P3 in the immunohistochemical analysis could be the use of formalin fixed brain samples and not cryosections as described which could interfere with intermediate filament protein detection [32]. Gfapδ staining P10 and P70 was evident in proximity to the ventricles and was similar to Gfapδ staining patterns in the adult human brain [11], [22], [29], [30], [31] and in the adult mouse brain [23], [32]. The Gfapδ positive regions were also positive for Gfapα, shown by a pan-Gfap antibody, but this pan-Gfap staining was also present throughout other brain regions in accordance with a general astrocyte staining. The lack of detectable Gfapδ staining in such cells could be a consequence of sensitivity according to the relative low level of Gfapδ expression. Vimentin and Nestin staining was only incompletely overlapping in the Gfapδ positive regions. The lineage and function of Gfap positive astrocytes and ependymal cells of the postnatal SVZ have been carefully described [45], [46]. Radial glial cells transform into Gfap positive cells and ependymal cells in the SVZ and astrocytes at other locations. The Gfap positive cells are progenitors for neuroblasts and glioblasts. The glioblasts can differentiate into astrocytes or oligodendrocytes during development and following brain injury. In many ways, ependymal cells resemble astrocytes: they express Gfap, are derived from radial glial transformation, and maintain glycogen as a functional energy store. However, they are unique in that they possess cilia and do not contact neuroblasts. We found that Gfapδ is highest expressed in the SVZ in the mouse post-natal brain. This is largely concordant with other observations concerning Gfapδ [11], [22], [23], [29], [30], [31]. Recently it was shown that all astroglia cells in the developing and adult mouse brain express Gfapδ regardless of the neurogenic potential indicating that in mice Gfapδ is not a neural stem cell marker as in humans [32]. Our studies, together with previous observations, suggest that Gfapδ positive cells always express a high level of Gfapα and that the relative expression ratio is constant [20], [21], [23], [27], [31], [32]. That Gfapδ was detected only in a subset of the pan-Gfap positive cells could be a matter of detection sensitivity.

Much attention has been denoted to understanding how intermediate filaments are regulated during cell growth, migration and morphology changes. Transcriptional regulations, post-translational modifications, capability to assembly into filaments, and protein localization have been carefully addressed. Moreover, it has been more and more established from studies in various cell models that mRNA localization regulates local protein synthesis during cell growth and migration. Many localized mRNA species have been described and their localization identified in several cell types [42], [47], [48]. Only few studies have described localization of mRNA for intermediate filament proteins in astrocytes. Dahlstrand et al. demonstrated subcellular localization of the *nestin* mRNA in processes of pial end-feet of radial glial cells by in situ hybridization assay on tissue sections from embryonic E10 mouse brains [49]. Other studies have demonstrated localization of *Gfap* mRNA in the branch points and distal parts of astrocyte protrusion by *In situ* hybridization studies on cultured rat type-2 astrocytes and in Müller cells of the rat eye retina [43], [50]. We here show that the different 3′-exon sequences included in *Gfapδ* and *Gfapα* mRNA determines different subcellular mRNA localization patterns. The *Gfapα* mRNA is present at a higher relative level in cell processes compared to the cell body if compared to the *Gfapδ* mRNA. It is important to note that only a very minor fraction of the total amount of *Gfapα* mRNA is localized to protrusions. However, the ratio between *Gfapα* and *Gfapδ* mRNA will be different in astrocyte protrusions compared to cell bodies by this different mRNA localization capability. Whereas the general expression ratio between *Gfapα* and *Gfapδ* mRNA is constant during development and in different astroglia cell types, variations in ratios between the different *Gfap* mRNA isoforms can still exist at the subcellular level. The differences in mRNA localization are determined by mRNA sequences present in the distal part of the transcripts and in accordance with the general pattern for RNA localization *cis*-elements to be located in the 3′-UTR [42], yet, the exact mechanisms involved in the localization of *Gfapα* mRNA need further examination. In general, Gfap expression is gradually up-regulated as Nestin and Vimentin expression decreases during brain development. Vimentin and Nestin containing intermediate filaments have potential to act as scaffolds for the establishment of long term Gfap intermediate filaments [1], [49]. It is straightforward to make a simple model wherein Gfap filaments within astrocytes initially can be locally synthesized at specific subcellular regions as a consequence of mechanisms which at least to some extend includes specific mRNA localization, and during astrocyte maturation, Gfap filaments are expanded and become more uniformly distributed throughout the cytoplasm. In such a scenario it can be important to decrease the relative concentration of the Gfapδ isoform at specific intracellular localizations due to specific functional properties of Gfapδ in terms of filament forming capacity and interaction with other proteins [21], [23], [26]. This could generate Gfap intermediate filaments with distinct surface structures and accordingly functional capacities at specific subcellular regions within astrocytes. How *Gfap* gene alternative splicing and associated differences in mRNA localization is linked to functional consequences for the intermediate filament dynamics will require further studies.

## Supporting Information

Table S1 **Sequences of FISH probes for** ***Gfapδ*** **and** ***Gfapα*** **mRNA.**
(DOC)
