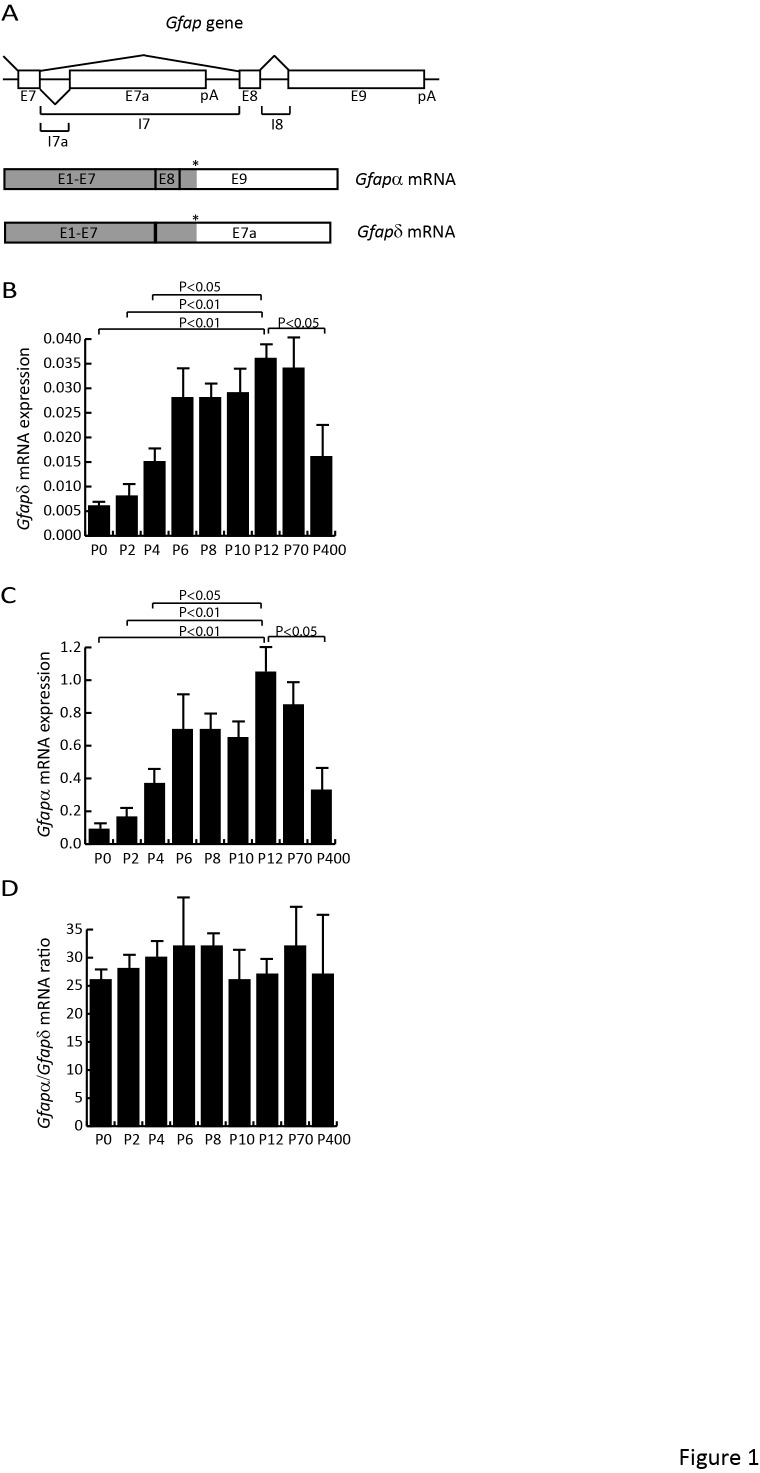# Correction: Alternative mRNA Splicing from the Glial Fibrillary Acidic Protein (*GFAP*) Gene Generates Isoforms with Distinct Subcellular mRNA Localization Patterns in Astrocytes

**DOI:** 10.1371/annotation/5b23b7fc-e140-471e-ac62-c0f59bfcc337

**Published:** 2013-11-06

**Authors:** Rune Thomsen, Tina F. Daugaard, Ida E. Holm, Anders Lade Nielsen

The version of Figure 1 in the published article is incorrect. The correct version of the Figure is available here: 

**Figure:**