# Supplementary material for: Alternative mRNA Splicing from the Glial Fibrillary Acidic Protein (GFAP) Gene Generates Isoforms with Distinct Subcellular mRNA Localization Patterns in Astrocytes
Source: PLoS One. 2013 Aug 26;8(8):e72110. doi: 10.1371/journal.pone.0072110 (PMC3753360; doi:10.1371/journal.pone.0072110)
Supplement: Table S1 — Sequences of FISH probes for Gfapδ and Gfapα mRNA. (DOC) [file pone.0072110.s001.doc]

Supporting Information Table S1

| **probe** | **Sequence** |
| --- | --- |
| *Gfapδ *#1  *Gfapδ *#2  *Gfapδ *#3  *Gfapδ *#4  *Gfapδ *#5  *Gfapδ *#6  *Gfapδ *#7  *Gfapδ *#8 | **T**GAGAGGTCT**T**GTGACTTTT**T**GGCCTTCCCC**T**TCTTTGGTGCTTT**T**GCCC  GC**T**CCATTTTCAA**T**CTGGTGAGCC**T**GTATTGGGACAAC**T**TGTATTGTGAG  **T**CTATCTAAGGGAGAGC**T**GGAGCACGCA**T**CTATCTAAGGGAGAGC**T**GGCA  A**T**CTATCTAAGGGAGAGC**T**GGAGCACGCC**T**CTATCTAAGGGAGAGC**T**GGA  **T**CTATCTAAGGGAGAGC**T**GGAGCACGCC**T**CTATCTAAGGGAGAGC**T**GGAG  C**T**AGCTCTATCGG**T**ATAACCTAAT**T**ACACAGAGCCAGGACC**T**AGGGTGGG  **T**TCAGAGTA**T**CTCCTTGGAGC**T**CCCAGGGACT**T**GCTGCCTTTAACAT**T**GG  G**T**GAAGCAATAGAAC**T**CTGGGGACACA**T**GAATCAAACACAGAGCC**T**GCCC |
| *Gfapα *#1  *Gfapα *#2  *Gfapα *#3  *Gfapα *#4  *Gfapα *#5  *Gfapα *#6  *Gfapα *#7  *Gfapα *#8 | **T**CTTTACCACGA**T**GTTCCTCT**T**GAGGTGGCCTTC**T**GACACGGATTTGG**T**G  C**T**CACATCACCACG**T**CCTTGTGCTCC**T**GCTTCGAG**T**CCTTAATGACC**T**CA  G**T**GAAGGGTATA**T**GCCATCTA**T**GAAGCTTAGCAC**T**GATTGAGCCT**T**GGGC  C**T**CTCTACTCTGC**T**CATCTTTCC**T**CTTCCCTTCCAA**T**TCTAACCCAGC**T**G  CCC**T**CTCCACTTA**T**GCCATAGATCC**T**GCCAAGTGC**T**GAGAACCAAGT**T**TC  AC**T**TTCAACTCCAG**T**GTCCAAATGA**T**CTCTACCAG**T**GCCTCTGCCAT**T**GG  G**T**CCCTCTCTCC**T**GTTTCAGTGTC**T**TCCAGAGAACGGG**T**TATTTCCT**T**GA  AC**T**GTGCAGCCAGGAA**T**AGACCTTCACAAC**T**GAGACACA**T**CTGTACCC**T**G |

Amino allyl modified thymine nucleotides are shown in bold.
